# Difference of Airborne Particulate Matter Concentration in Urban Space with Different Green Coverage Rates in Baoji, China

**DOI:** 10.3390/ijerph16081465

**Published:** 2019-04-25

**Authors:** Ling Qiu, Fang Liu, Xiang Zhang, Tian Gao

**Affiliations:** College of Landscape Architecture and Arts, Northwest A&F University, Xianyang 712100, Shaanxi, China; qiu.ling@nwsuaf.edu.cn (L.Q.); lf472811832@163.com (F.L.); 18710884631@163.com (X.Z.)

**Keywords:** PM2.5, PM10, ecosystem services, vegetation structure, landscape management

## Abstract

With the acceleration of urbanization and industrialization, the problem of airborne particulate pollution has become more and more serious. Green areas in urban spaces with different green coverage rates in Baoji City were selected to quantitatively compare the effects and differences of month, time, temperature, humidity, wind velocity, vegetation structure, and area of site on PM2.5 and PM10 concentration. The results showed that increasing the urban green coverage rate will help to improve the green area’s reduction of airborne particulate matter concentration and the selected factors affecting the green area’s reduction ability were discrepant in urban spaces with different green coverage rates. With the decrease of the green coverage rate, the purification effect of green area itself on air particles was weakened, and other factors, such as meteorological conditions and human activities, became the dominant influencing factors. Vegetation structure only had significant effects on the concentration of PM2.5 and PM10 in green areas of urban space with a green coverage rate greater than 75%. The concentration of PM2.5 and PM10 were lowest in the partly closed green area of one-layered coniferous trees and the closed green area of one-layered mixed trees. The research shows that green areas in urban spaces with different green coverage rates have different reduction effects on the concentration of airborne particles, which provides a theoretical basis and reference for the optimization of green area structures and to improve air quality effectively in the future.

## 1. Introduction

With the acceleration of urbanization in China, the problem of airborne particulate pollution is becoming more and more serious [1,2]. China’s Ministry of Eco-Environment issued the “2017 China’s Ecological Environment Bulletin”, which showed that 239 cities did not meet the environmental air quality standards in 2017, accounting for 70.7% of the total number of cities, with the overall situation not being optimistic. As we know, PM10 is one of the primary pollutants that affects air quality and damages human health through the respiratory tract [3]. Smaller PM2.5 is even more deleterious, and can enter the alveolar and the blood circulation system, causing a variety of human systemic diseases [4,5].

As the basic part of an urban ecosystem, urban green space plays an important role in improving environmental quality, beautifying the environment, regulating the urban microclimate, and promoting urban sustainable development [6,7]. However, due to the dispersal and fragmentation of urban green spaces in the process of rapid urbanization, whether or not the current urban landscape pattern can exert a considerable influence on ecological ecosystem services is unknown [8]. Several studies showed that urban areas with a high green coverage helped to reduce the concentration of airborne particulate matter and often had a negative correlation with it [9,10]. Selmi et al. (2016) found that different land use types, such as forests, parks, industrial areas, and residential areas, had different effects on the reduction of airborne particulate matter, which was related to the ratios of tree coverage of each specific land use type [11]. However, the range of the green coverage rate in urban spaces in which the role of green areas in purifying air can be effectively exerted and which factors affect the concentration of airborne particulate matter in such green areas have rarely been studied.

As the main body of urban green spaces, plants play a major role in improving air quality. This can be considered from two angles. The physiological and ecological characteristics of the plant itself can play a certain role. The combination of different plants in green spaces also produces different ecological benefits. The impact of plants itself on air particle deposition depends on the scale considered [12]. Considering the individual leaves of the plant, different surface structures, such as a rough surface, thicker waxy layer, and leaf hair, were identified as being more helpful in capturing airborne particles [13,14,15,16,17]. From the perspective of individual plants, broadleaf species with rough foliage have a higher ability to capture particulate matter than smooth foliage species; conifers that produced a thicker layer of epidermal wax were found to be more effective in accumulating particulate matter than broadleaf species, and evergreen conifers were likely to accumulate contaminants throughout the year [18,19,20,21]. However, these studies simply focused on the role of individual plants, and, in fact, the ecological effects of green space are cumulatively produced by a variety of plant combinations. Considering certain plant species may not be comprehensive enough, and as such, the present study considered the whole green space as the research object, taking into account the overall reduction of air particles by the green space.

Vegetation structure, as an important factor reflecting the spatial and temporal distribution characteristics and the dynamic changes of green space ecological functioning, has seldom been studied systematically to explore the ability of green spaces and different vegetation structures’ effects on reducing the concentration of airborne particles. Gao et al. (2015) showed that green spaces with complex structures have better effects than ones with simple structures, such as lawns and single shrubs, in reducing airborne particulate matters [22]. Conversely, Nguyen et al. (2015) demonstrated that the concentration of airborne particulate matter in green spaces enclosed by lawns and shrubs was lower than that in a composite structure of green space [23]. Some studies even found that there were no differences in the reduction of airborne particulate matter in different communities of green spaces [24]. These inconsistencies require further attention to expose the underlying factors. The lack of a unified standard of the vegetation structure classification of green space as well as a comprehensive consideration of environmental factors and site-specific features could be the primary explanation [10,22]. 

Therefore, this study took green areas in an urban space with different green coverage rates in Baoji City as study areas and compared the effects and differences of the month, time, temperature, humidity, wind velocity, vegetation structure, and area of the site on the concentration of PM2.5 and PM10 quantitatively. Through this study, the green coverage range of an urban space in which a green area can effectively exert its ability to improve air quality was determined, as well as which type of vegetation structure works best. This provides a theoretical basis and practical methods for the optimization of urban overall green space planning and green area vegetation planting design in the future.

## 2. Materials and Methods

### 2.1. Study Area

Baoji is situated in the western part of the Guanzhong Plain in Shaanxi province, China between 106°18′ E, 108°03′ E and 33°35′ W, 35°06′ W, belonging to the semi-humid climate zone of mid-latitude temperature climate. The lowest temperature is in January during which time the average minimum temperature is −3.5 °C. The highest temperature is in July during which time the average maximum temperature is 30.9 °C. The annual average precipitation is 700 mm [25]. The city is surrounded on three sides by mountains, and the Weihe River passes through the center of the city.

**Figure 1:**
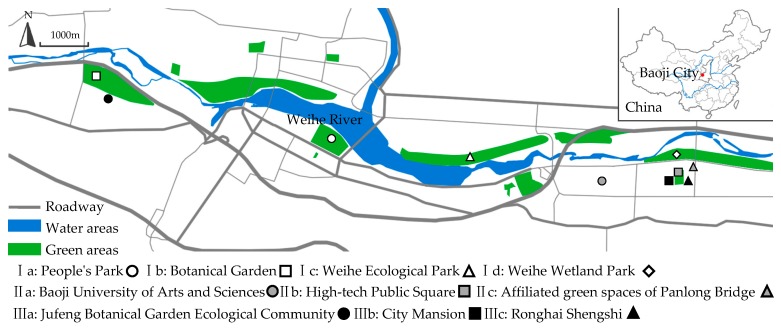
Location of the study sites (I–III).

The study area was selected along both sides of the Wei River within the city of Baoji, covering an area of 35.39 km^2^, accounting for 40% of the total built area, comprising of a variety of urban habitats, and was subject to an investigation of a new green belt for ecological regulating use (Figure 1).

### 2.2. Categorization of Urban Spaces by Green Coverage Rate

The urban space was divided into three urban landscape patterns according to the green coverage rate, which was based on the analysis of aerial photos of Baoji’s built-up area derived from National Gaofen-2 satellite and the characteristics obtained through field research of the green area in Baoji City: I (>75% of green coverage ratio), II (35%–75% of green coverage ratio), and III (<35% of green coverage ratio). The space with >75% of a green coverage ratio referred to urban forests and urban parks; urban space with a green coverage rate ranging from 35% to 75% included the campus, square and affiliated green spaces next to the bridge across the river; the urban space with a green rate of less than 35% referred to the urban residential areas (Figure 1).

### 2.3. Construction of a Green Area Classification System Incorporating Vegetation Structure Factors

According to the characteristics of Baoji’s urban landscape patterns, a three-level urban green area classification system integrated with vegetation structure parameters, including the vegetation horizontal structure, vertical structure, and forest type, was constructed [26,27]. The first level was divided into open green area (<10% canopy cover of trees/shrubs), partly open green area (10%–30% canopy cover of trees/shrubs), partly closed green area (30%–70% canopy cover of trees/shrubs), and closed green area (>70% canopy cover of trees/shrubs) according to the horizontal structure of the vegetation; the second level was divided into broad-leaved forest, coniferous forest, and broad-leaved and coniferous mixed forest based on the forest type; the third level mainly focused on the division of the vertical structural features of the green area. In this study, one-layered structures only contained a tree layer, more than one-layered structures referred to double tree layers or tree layer and shrub layer combinations (Table 1).

**Figure 2:**
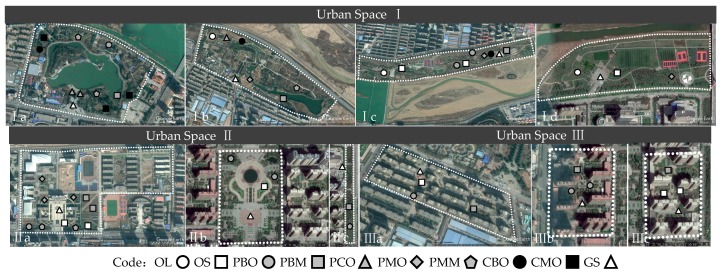
Distribution of nine different vegetation structure plots and their repetition in urban space with three different green coverage rates.

Based on the above classification system and the actual situation of the vegetation structure types in Baoji city, 51 green areas of 9 different vegetation structures in three different green coverage rate urban spaces were finally selected as sample plots. The repetition and distribution of each type of green area was as follows (Figure 2 and Table 2). The surrounding environment of the 10 research sites was similar, with no obvious sources of pollutants, such as factories and highways. A hard control (GS) was set in each site in the urban space with different green coverage rates to ensure that every urban space included both the control groups and the green areas as a comparison.

### 2.4. Monitoring of Airborne Particle Concentration

The concentrations of PM2.5 and PM10 and the meteorological factors, including wind velocity, temperature, and humidity, were tested at the same time using a hand-held particle counter (Aerocet 831) and a hand-held weather station (FC-36025). The instruments were zeroed before each monitoring. The area calculation of each sample plot was measured by using a hand-held GPS receiver (Garmin GPS map 629sc) to obtain the latitude and longitude around the sample plots, and then the plot coordinates were introduced into ArcGIS 10.2 software combined with the satellite image to calculate the area accurately.

The on-site monitoring was carried out for one year from April 2017 to March 2018. In order to avoid dramatic changes in the meteorological factors, the concentrations of PM2.5 and PM10 were tested in each sample plot every two hours, five times a day from 8 a.m to 6 p.m in good weather conditions within three days every month. Two typical vegetation structures located in each of the plots were selected as the sampling point. During the same period of time, the test team members monitored all the samples in each site in the same sequence at the same time. All the tests were performed at a height of 1.5 m, which is the average height of human respiration.

### 2.5. Data Analysis

In this study, Microsoft Office Excel 2007 software (Microsoft Corporation, Redmond, WA, USA) was used for all data recording and collection. Generalized regression analysis was used by the statistical software package, Minitab 16 (State College, PA, USA), to model all variables in the experiment, including independent variables, such as the green coverage rate, month, time, wind velocity, temperature, humidity, vegetation structure, and area, and dependent variables, including PM2.5 and PM10 concentrations [28]. This included the analysis of variance, which first explored whether the variables in this study had a significant effect on airborne particulate concentrations, followed by an analysis of how each variable affected the concentration of airborne particulate matter by generalized regression analysis. The acceptable significance level was *p* < 0.05.

## 3. Results and Discussion

Variance analysis of all variables in the study found that all factors had extremely significant effects on PM2.5 concentration (*p* < 0.01, *R*^2^ = 71.39%). Except for wind velocity and area, the other factors also had significant effects on PM10 concentration (*p* < 0.01, *R*^2^ = 67.25%) (Table 3).

### 3.1. Effects of Different Green Coverage Rate of Urban Spaces on PM Concentration

The PM2.5 and PM10 concentrations in the selected green areas of three urban spaces with different green coverage rates were significantly discrepant, and their reductions in airborne particulate matter were ranked as urban space I > urban space II > urban space III (Table 4), indicating the green coverage rate was negatively correlated with airborne particulate matter concentration.

The factors affecting the concentration of airborne particulate in green areas of three urban space with different green coverage rates were not identical. In urban space I, all variables had a significant effect on the PM2.5 concentration, but wind velocity and area had no significant effects on the PM10 concentration. With the decrease of the green coverage rate, the influence of the vegetation structure and area on the PM2.5 concentration was weakened to no significant effect in urban space II, and in addition to wind speed and area, the effect of the vegetation structure on the PM10 concentration was also reduced to no significant effect. In urban space III, there was still no significant effect of the vegetation structure and area on the PM2.5 concentration. For the PM10 concentration, there was no significant effect on time, temperature, wind velocity, vegetation structure, and area (Table 5).

The results showed that the urban green coverage rate had a significant effect on the concentration of PM2.5 and PM10. At different scales, the intrinsic properties of the vegetation and external factors worked together to influence the concentration of airborne particulate matter. The green areas in urban space I with the highest green coverage rate (>75%) had the best effect on the reduction of airborne particulate matter, which was consistent with the findings of previous studies [10,11]. Only in the background environment of the urban space with a high green coverage rate was the influence of green characteristics, such as vegetation structure and area, on particulate matter concentration dominant [29]. At the same time, the influence of meteorological factors and time factors on the concentration of particulate matter was also significant. However, as the green coverage rate decreased, the types and strengths of the airborne particle concentrations affected by the selected variables in this study were reduced. In the urban space with a middle green rate (75%–35%), the impact of green area characteristics weakened, but meteorological factors and time factors still had significant effects. The main reason for this was possibly due to the reduction of green areas. In the urban space with the lower green rate (<35%), the effects of meteorological factors and time factors was also reduced to some extent, which may be the result of the large gray space of the outside environment causing a large disturbance, such as the most common car exhaust emissions in cities and the fumes produced by urban inhabitants in their daily lives [30]. Based on the above results, it is implied that increasing the green coverage rate and the use of a vegetation removal mechanism could be a more efficient way to reduce the pollution of urban air particles in contexts where completely reliance on the control of pollution sources is not possible, and to reduce the generation of airborne particulate matter from the source [31,32].

### 3.2. Effects of Time Factors on PM Concentration

**Figure 3:**
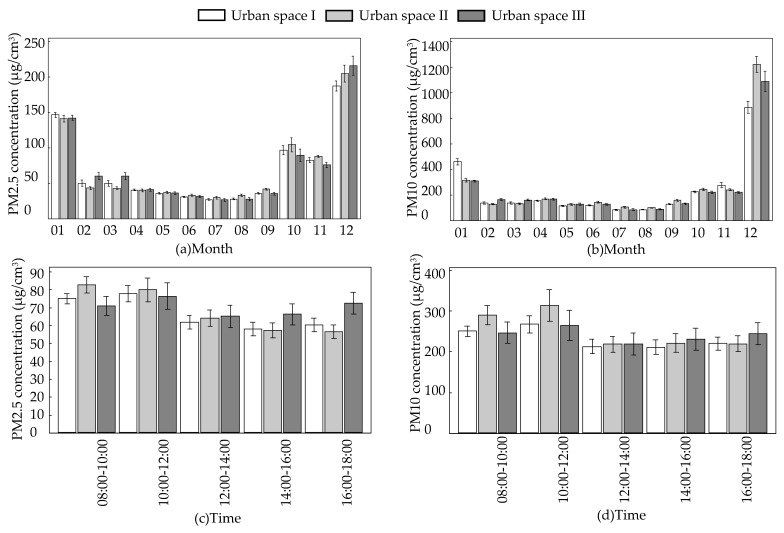
The relationship between time factors and PM concentration. (**a**) The relationship between month and PM2.5 concentration; (**b**) the relationship between month and PM10 concentration; (**c**) the relationship between time and PM2.5 concentration; (**d**) the relationship between time and PM10 concentration.

The monitoring month had a significant effect on the concentration of PM2.5 and PM10 in the green areas of three urban spaces with different green coverage rates, and the concentrations of airborne particulates followed the same trend with as the monthly changes. The concentrations were lowest in July and August during the summer and the highest in December and January during the winter (Table 5, Figure 3a,b).

Airborne particulate concentrations were subject to a variety of factors that could produce very significant seasonal changes. During the summer, plant growth enters a vigorous period in which the leaves grow completely and the metabolism is higher, so the dust retention capacity is higher and the concentration of particulate matter in the air is lower. During winter, most plant leaves wither, grow slowly, and enter a dormant period, so the dust retention ability is the weakest during this time, and thus, concentrations of PM2.5 and PM10 reached the highest values in this period [11,22,23,33,34]. Affected by meteorological conditions, the scale of air diffusion during the summer is large, and the particles are easily diffused to a high altitude, so concentrations of particulate matter are low. The scale of air diffusion during the winter is small, and the particles are not easily diffused to high altitudes, so the concentration of particulate matter is high [35,36]. In addition, the higher concentration of particulate matter during winter is also related to the winter heating period in northern cities. Increases in coal combustion increase the concentration of airborne particulate matter [34,36]. The monthly changes in the green areas of different green coverage rate urban spaces in this study had the same significant effect on both PM2.5 and PM10 concentrations and the trend was consistent, which was related to the physiological state of vegetation and the change of external meteorological conditions with the change of the month. However, the concentration of airborne particulates in the green areas of an urban space with a high green coverage rate was generally low, which indicates that the green area could play a more effective role in dust retention in the background environment with a high green coverage rate urban space. In the low green coverage environment, a small proportion of green area could not exert maximum ecological benefits, and was affected by many other factors, such as meteorological factors or human activities in a large proportion of hard areas.

The change in time during the day had no significant effect on PM10 concentration in green areas of urban space III, but had a significant effect on PM2.5 and PM10 concentrations in green areas of urban space I and II. As the time of day changed, the airborne matter concentration followed the same trend, which was higher in the morning, highest at noon, lower in the afternoon, and tended to be stable (Table 5, Figure 3c,d).

The concentration of airborne particle changes with the time of day were mainly due to a combination of human factors and meteorological factors [37]. Although there was no obvious source of pollutants, such as factories and boilers, around the study area, vehicle exhaust emissions and road dust still existed, and the airflow exchange after sunrise strengthened, which may have resulted in an air particulate matter concentration increase during the peak period of commuting at noon. During the afternoon, traffic volumes and flows of people were relatively small, pollution was light, and the diffusion of particulate matter was enhanced, so the concentration of airborne particles was low [22,24]. Previous studies have shown that air particulate matter concentration is positively correlated with human flow and traffic volume [38,39]. Automobile exhaust emissions have a great impact on air particulate matter concentrations [40]. The adoption of relevant measures for strict control could effectively reduce airborne particulate matter concentrations [41]. The concentration of airborne particulate matter in green areas of urban space with high coverage was generally low. In urban space III, which had a low green coverage rate, the large-scale surrounding gray space may have generated great pollutants, which could not be effectively absorbed by the small proportion of green area. The improvement of the meteorological conditions by small green areas was also very limited, which was not conducive to the settlement of particulate matter. In addition, the larger the particle sizes, the worse the absorption of the vegetation [42]. A small area of green space could adsorb small particles to a certain extent, but it was powerless for large particles. That is why the change in time during the day had no significant effect on the PM10 concentration in urban space III.

### 3.3. Effects of Meteorological Factors on PM Concentration

**Figure 4:**
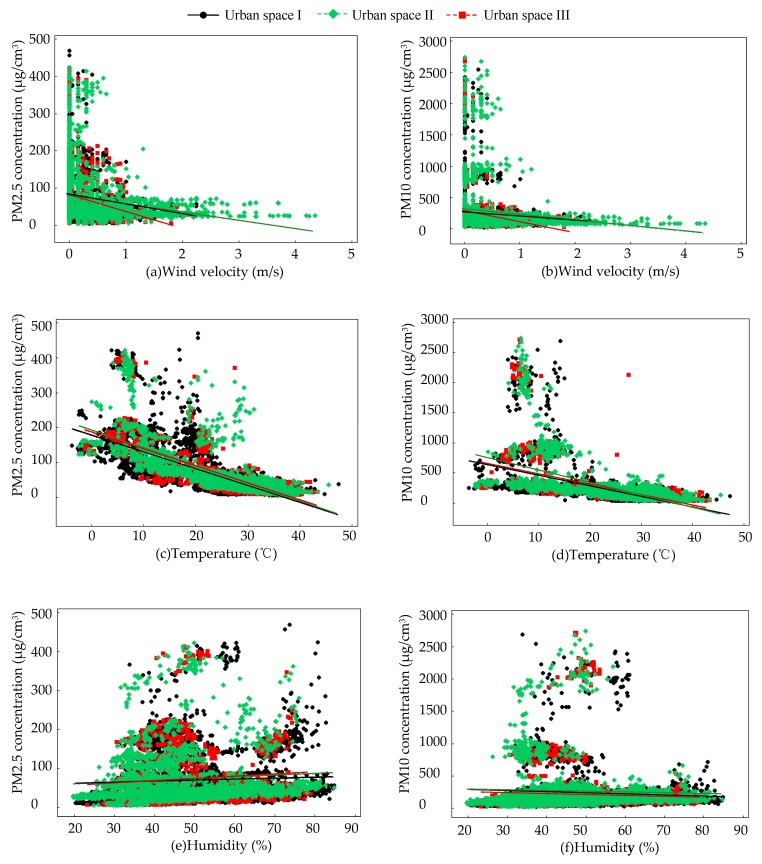
The relationship between meteorological factors and PM concentration. (**a**) The relationship between wind velocity and PM2.5 concentration; (**b**) the relationship between wind velocity and PM10 concentration; (**c**) the relationship between temperature and PM2.5 concentration; (**d**) the relationship between temperature and PM10 concentration; (**e**) the relationship between humidity and PM2.5 concentration; (**f**) the relationship between humidity and PM10 concentration.

Wind velocity had a negatively significant effect on PM2.5 concentration in the green areas of three different landscape patterns of urban spaces, but had no significant effect on the PM10 concentration (Table 5, Figure 4a,b). This study chose sunny and breezy weather for field monitoring with the aim of avoiding dust and secondary dust on the ground caused by an excessive wind velocity and turbulence, which in turn would affect the airborne particulate matter concentration. Some studies have found that the concentration of airborne particulate matter increased first and then decreased with the increase of wind velocity [43]. The wind velocity was too small to fully diffuse the particulate matter to increase its concentration. As the wind velocity increased, the particle diffusion increased and the concentration decreased. However, some studies have shown that the concentration of particulate matter in gale weather was at a low level, but in addition to gale days, PM2.5 concentration was still negatively correlated with wind velocity [44]. Wind velocity had the greatest influence on fine particles with a smaller particle size and was negatively correlated with its concentration by affecting the horizontal diffusion of particulate matter [45]. Although wind velocity had a negative significant impact on PM2.5 concentration in all green areas, the influence of wind velocity on PM2.5 concentration in green areas increased with the decrease of the green coverage rate of the urban space where the green area was located. This can be seen from the coefficients of the fitted regression equation (urban space I: PM2.5 = 70.00–21.55 wind velocity, urban space II: PM2.5 = 78.23–21.83 wind velocity, urban space III: PM2.5 = 76.46–41.89 wind velocity). The lower the green coverage rate is, the greater the influence of the wind velocity on the concentration of airborne particles in the green area. The lowering of the retardation of plants in a small proportion of the green areas was more conducive to carry and transport the airborne particulate matter by the wind. Overall, the concentration of airborne particulate matter in green areas of an urban space with high green coverage rate was low, indicating that the impact of the green coverage rate on airborne particulate matter was greater than wind velocity. However, several studies showed that the wind direction has divergent effects on the concentration of airborne particles [10,36]. This should be explored in a future study.

Temperature had no significant effect on the PM10 concentration in green areas of urban space III, but it had a negatively significant effect on the PM2.5 and PM10 concentration in all the other green areas (Table 5, Figure 4c,d). As the temperature increased, the convection of the atmosphere in the vertical direction was more frequent. Such gas circulation exchange accelerated the transportation of airborne particles and helped to reduce the concentration of airborne particulate matter [37,44]. The increase in temperature was also conducive to the formation of secondary aerosols, which was negatively correlated with PM2.5 and PM10 concentrations [45]. As the temperature increased, the photosynthesis of plants strengthened, which helped to enhance the ability of green plants to absorb airborne particles [46]. In urban space III, which had a low green coverage rate, the absorption of particulate matter by the small-scale green area was weakened, especially for coarse particulate matter, PM10. In the urban space with a green coverage rate of more than 75%, temperature had the least effect on airborne particles, which can be seen from the coefficients of the fitted regression equation (urban space I: PM2.5 = 177.80–4.85 temperature, PM10 = 632.6–17.51 temperature, urban space II: PM2.5 = 190.5–5.07 temperature, PM10 = 747.00–20.60 temperature, urban space III: PM2.5 = 185.60–4.88 temperature). Although temperature had a stronger effect on the reduction of airborne particulate matter concentration in the green area of the urban space with a low green coverage rate, overall, the concentration in the green area of urban space with a high green coverage was low, indicating that the impact of the green coverage rate was greater than the temperature.

Humidity had a significant effect on the PM2.5 and PM10 concentration in the green areas of three different landscape patterns of urban spaces, but there were some differences between different particulate sizes (Table 5, Figure 4e,f). From the regression fitting equation, it can be seen that the correlation between humidity and PM2.5 and PM10 was opposite (urban space I: PM2.5 = 54.42 + 0.27 humidity, PM10 = 320.50–1.76 humidity, urban space II: PM2.5 = 50.80 + 0.43 temperature, PM10 = 307.00–1.11 temperature, urban space III: PM2.5 = 45.31 + 0.56 temperature, PM10 = 252.90–0.26 temperature). The concentration of PM2.5 was positively correlated with humidity, but the PM10 concentration was negatively correlated with humidity. Within a certain range, fine particles were more likely to act as condensation nuclei and then absorb moisture, with the relative humidity increased to increase PM2.5 concentration [37,44]. Humidity affected the concentration of particulate matter because of its hygroscopicity, and the size of the particles also affected its sedimentation [47,48]. The coarse particulate matter, PM10, absorbed and swelled as the humidity increased, but the larger particle size was more favorable for its sedimentation [36,49]. In addition, the increase in wettability and relative humidity may cause certain emission mechanisms of biological particles, such as active squirting of fungal spores or flower pulverization caused by hygroscopic swelling, and may also increase the concentration of atmospheric particulate matter around plants to some extent [50]. Although the effect of humidity on particles of different sizes was different, the effect of humidity on the increase of the PM2.5 concentration was the smallest, and the decrease of the PM10 concentration was the largest in the green areas of the urban space with a high green coverage rate. Overall, in the urban space with a high green coverage rate, a large proportion of green area and humidity played a positive role in the reduction of the airborne particulate matter concentration.

### 3.4. Effects of Characteristics of Green Area on PM Concentration

**Figure 5:**
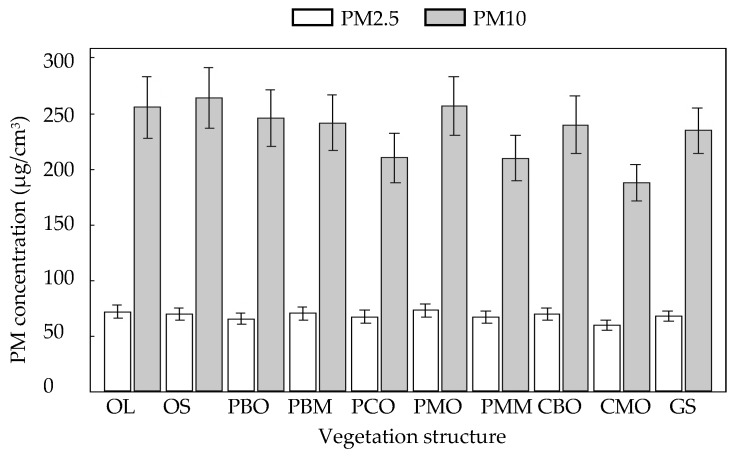
The relationship between vegetation structure and PM concentration in urban space I.

The vegetation structure only had a significant effect on the concentration of PM2.5 and PM10 in green areas of urban space I, but had no significant effect on the other two types of urban spaces (Table 5). Among them, higher concentrations of PM2.5 in OL, OS, PMO, and GS; lower concentrations of PM2.5 in PCO and CMO; higher concentrations of PM10 in OL, OS, and PMO; and lower concentrations of PM10 in PCO, PMM, and CMO were found. This is evident from Table 6, while Figure 5 shows that the differences between different vegetation structures could be broadly seen, but some places were not very obvious, probably due to the range of confidence intervals (95%).

This study systematically classified urban green area from the three levels of the horizontal structure, forest type, and vertical structure of green areas. According to the results of comprehensive data analysis, it was only in urban space I, which had a high green coverage rate, that the vegetation structure had a significant impact on the concentration of airborne particulate matter. In the other two types of urban space, a large proportion of surrounding gray area may have caused greater interference, and the green area’s purification effect on air particles was reduced. Only in the condition of high vegetation coverage and good vegetation growth can the air particulate concentration be effectively reduced [29]. Among them, the open green area dominated with lawn (OL) and shrubs (OS) and the partly closed green area of one-layered mixed trees (PMO) had higher concentrations of PM2.5 and PM10, while the partly closed green area of one-layered coniferous trees (PCO) and the closed green area of one-layered mixed trees (CMO) had lower concentrations of PM2.5 and PM10. The airborne particles in the open green area were less affected by vegetation, but were instead affected by meteorological factors, such as wind speed and humidity [51]. The particulate matter absorbed on the plant surface was only temporarily retained, which was highly susceptible to meteorological factors and thus easily rebounded into the atmosphere, thereby increasing the concentration of airborne particles [32,52]. The partly closed green area of one-layered coniferous trees (PCO) and the closed green area of one-layered mixed trees (CMO) had more and higher coverage vegetation than the open green area, which was conducive to the accumulation of air particles. Compared with pure broad-leaved forests, thicker epidermis wax layers or surface secretions of conifer species were more effective in retaining airborne particulate matter, and the accumulation of particulate matter could occur throughout the whole year [18,19,20,21,42]. Compared with more-than-one-layered forests, one-layer forests were beneficial to the circulation of air to a certain extent, ensuring gas exchange inside and outside the forest, thereby significantly reducing the concentration of airborne particulate matter.

The area was also an important factor in measuring the ability of a green area to retain dust. The results of this study showed that the area only had a significant effect on the PM2.5 concentration in green areas of urban space I with the area ranging from 0.1 to 2.3 hectares. The existing research also showed that the higher the green coverage rate and the greater the green area, the better the reduction of particulate matter in urban forests and parks [11]. In the other two types of urban spaces with low green coverage rates in this study, the green area was too small to significantly reduce the concentration of particulate matter, and large areas of gray space may have generated a large amount of pollutants. Wang et al. (2014) found that more than 50 hectares of green space in a park could reduce the concentration of PM2.5 in the air [53]. Although the area had a significant influence on the concentration of PM2.5 in this study, it was not very strong, and the regression coefficient of the fitting equation was small (PM2.5 = 69.58–0.000305 area). It may be that the range of the area change was not large enough, and the dust-retaining effect of larger green areas should be explored in the future. In addition, the increase of the green area also promoted a decrease of the PM10 concentration in the air [33,54], but the specific critical value was been identified in this study, which only showed that the smaller the size of the particles, the more significant the absorption of vegetation. Further exploration is thus needed.

## 4. Conclusions

In this study, the green areas of urban spaces with different green coverage rates in Baoji City were used as the research areas. The effects of the month, time, temperature, humidity, wind velocity, vegetation structure, and area on the concentration of PM2.5 and PM10 were quantitatively compared. A variety of factors worked together to change the concentration of airborne particles in the green areas, but the dominant factors were different in urban spaces under different green coverage rates

The higher the green coverage rate of the urban space, the more significant the influence of the variables selected in this study on the concentration of airborne particles in green areas, especially the characteristics of green areas, such as the vegetation structure and area. The partly closed green area of one-layered coniferous trees and the closed green area of one-layered mixed tree vegetation structure types had the greatest reduction effect on PM2.5 and PM10 concentrations. As the green coverage rates decreased, the airborne matter concentration was more disturbed by the external gray area. The impacts of the characteristics of green areas were weakened to no influence, but the influence of meteorological factors and time factors on the concentration of airborne particulate matter was still significant, especially the impact of humidity and monthly changes.

The above results showed that increasing the urban space green coverage rate would help to fully utilize the green area’s ability to reduce of airborne particulate matter, and the study found that green areas with two types of vegetation structures have the best reduction effect. The two planting models could be prioritized in the planning and design of vegetation planting for the purpose of improving air quality in the future. In an urban space with low green coverage, a small proportion of green space may not have the desired ecological benefits. This has important implications for future urban green space planning and design, vegetation planting mix design, and green space ecological benefit research.

## Figures and Tables

**Table 1:** Classification of Baoji City green area with vegetation structure factors.

Level 1	Level 2	Level 3
Open green area (<10% canopy cover of trees/shrubs)	Lawn Shrub	-
Partly open green area (10%–30% canopy cover of trees/shrubs)	Broad-leaved Coniferous Mixed	One-layered More-than-one-layered
Partly closed green area (30%–70% canopy cover of trees/shrubs)		
Closed green area (>70% canopy cover of trees/shrubs)		

**Table 2:** Type, repetition, and distribution of vegetation structure in urban space with three different green coverage rates.

Vegetation Structures	Number of Green Areas in Each Different Green Coverage Rate Urban Spaces										
	Urban Space I (>75% of Green Coverage Ratio)				Urban Space II (35%–75% of Green Coverage Ratio)			Urban Space III (<35% of Green Coverage Ratio)			Total
	Ia	Ib	Ic	Id	IIa	IIb	IIc	IIIa	IIIb	IIIc	
OL ^1^		1	1	1							3
OS ^2^			2	1	2	1		1		2	9
PBO ^3^	1		2		1	2		1	2		9
PBM ^4^	1	1	1		2		1	1	1	1	9
PCO ^5^	2	1									3
PMO ^6^		1	1	1	3						6
PMM ^7^	2	1			2		1				6
CBO ^8^	1	1	1								3
CMO ^9^	3										3
Sum	10	6	8	3	10	3	2	3	3	3	51

^1^ OL: Open green area dominated with lawn; ^2^ OS: Open green area dominated with shrubs; ^3^ PBO: Partly closed green area of one-layered broad-leaved trees; ^4^ PBM: Partly closed green area of more-than-one-layered broad-leaved trees; ^5^ PCO: Partly closed green area of one-layered coniferous trees; ^6^ PMO: Partly closed green area of one-layered mixed trees; ^7^ PMM: Partly closed green area of more-than-one-layered mixed trees; ^8^ CBO: Closed green area of one-layered broad-leaved trees; ^9^ CMO: Closed green area of one-layered mixed trees.

**Table 3:** Variance analysis of factors affecting PM concentration.

Factors	Df ^1^	PM2.5		PM10	
		F ^2^	P ^3^	F ^2^	P ^3^
Green coverage rate	2	56.97	0.00	17.01	0.00
Month	11	578.27	0.00	1014.24	0.00
Time	4	9.78	0.00	37.91	0.00
Temperature	1	85.90	0.00	27.17	0.00
Humidity	1	1927.18	0.00	190.79	0.00
Wind velocity	1	27.97	0.00	0.01	0.91
Vegetation structure	9	11.67	0.00	9.91	0.00
Area	1	13.20	0.00	0.17	0.68

^1^ Df: Degree of freedom; ^2^ F: Variance test volume; ^3^ P: Significant test of regression equation.

**Table 4:** Generalized regression analysis of PM concentration and urban space with different green coverage rates.

Urban Space with Different Green Rate	PM2.5				PM10			
	C ^1^	SE ^2^	T ^3^	P ^4^	C ^1^	SE ^2^	T ^3^	P ^4^
Urban space I	−5.51	0.54	−10.18	0.00	−12.04	2.75	−4.37	0.00
Urban space II	3.42	0.59	5.78	0.00	14.70	3.01	4.88	0.00
Urban space III	2.09	0.69	3.03	0.00	−2.66	3.50	−0.76	0.45

^1^ C: Coefficient; ^2^ SE: Coefficient standard error; ^3^ T: Significant test of regression parameters; ^4^ P: Significant test of regression equation.

**Table 5:** Variance analysis of factors affecting PM concentration in urban space with different green coverage rates.

Factors	Df ^1^	Urban Space I				Urban Space II				Urban Space III			
		PM2.5		PM10		PM2.5		PM10		PM2.5		PM10	
		F ^2^	P ^3^	F ^2^	P ^3^	F ^2^	P ^3^	F ^2^	P ^3^	F ^2^	P ^3^	F ^2^	P ^3^
Month	11	229.20	0.00	324.70	0.00	232.44	0.00	679.04	0.00	167.57	0.00	298.01	0.00
Time	4	3.22	0.01	14.75	0.00	27.51	0.00	27.89	0.00	6.18	0.00	1.41	0.23
Temperature	1	22.63	0.00	82.80	0.00	62.33	0.00	30.10	0.00	9.77	0.00	0.23	0.64
Humidity	1	868.79	0.00	169.13	0.00	672.28	0.00	25.49	0.00	511.14	0.00	50.64	0.00
Wind velocity	1	40.54	0.00	0.24	0.62	9.63	0.00	0.14	0.71	49.69	0.00	3.00	0.08
Vegetation structure	9	10.98	0.00	9.77	0.00	0.86	0.51	0.91	0.48	0.64	0.59	0.37	0.77
Area	1	4.89	0.03	0.02	0.89	2.57	0.11	0.94	0.33	0.02	0.89	0.32	0.57

^1^ Df: Degree of freedom; ^2^ F: Variance test volume; ^3^ P: Significant test of regression equation.

**Table 6:** Generalized regression analysis of PM concentration and vegetation structure in urban space I.

Vegetation Structure	PM2.5				PM10			
	C ^1^	SE ^2^	T ^3^	P ^4^	C ^1^	SE ^2^	T ^3^	P ^4^
OL	5.70	1.46	3.91	0.00	22.45	7.41	3.03	0.00
OS	4.25	1.48	2.88	0.00	28.67	7.51	3.82	0.00
PBO	−1.74	1.45	−1.20	0.23	9.12	7.38	1.24	0.22
PBM	−0.52	1.49	−0.35	0.73	5.75	7.57	0.76	0.45
PCO	−5.59	1.55	−3.62	0.00	−30.69	7.86	−3.90	0.00
PMO	6.78	1.44	4.69	0.00	24.28	7.34	3.31	0.00
PMM	−2.20	1.64	−1.35	0.18	−26.17	8.33	−3.14	0.00
CBO	0.68	1.44	0.47	0.64	5.31	7.34	0.72	0.47
CMO	−10.00	1.45	−6.87	0.00	−44.00	7.40	−5.95	0.00
GS	2.65	1.30	2.03	0.04	5.30	6.63	0.80	0.42

^1^ C: Coefficient; ^2^ SE: Coefficient standard error; ^3^ T: Significant test of regression parameters; ^4^ P: Significant test of regression equation.
